# National mortality trends due to OSA and respiratory failure in U.S adults from 1999–2023: Insights from the CDC-WONDER database

**DOI:** 10.1097/MD.0000000000050662

**Published:** 2026-09-18

**Authors:** Maryam Saghir, Eshal Saghir, Nida Shoaib, Barka Sajid, Muhammad Affan, Hasibullah Aminpoor, Hafsa Shahid, Javeria Shamim Hayat, Syed Saddam Hussain

**Affiliations:** a Department of Medicine, Jinnah Sindh Medical University, Karachi, Pakistan; b Department of Medicine, Dow University of Health sciences, Karachi, Pakistan; c Department of Internal Medicine, Jinnah Sindh Medical University, Karachi, Pakistan; d Department of Medicine, Jinnah Sindh Medical University, Karachi, Pakistan; e Department of Medicine, Jinnah Sindh Medical University, Karachi, Pakistan; f Faculty of Medicine, Kabul University of Medical Sciences “Abu Ali Ibn Sina,” Kabul, Afghanistan; g Resident Physician, Department of Internal Medicine, Rabia Balkhi National Complex Hospital, Kabul, Afghanistan; h The MetroHealth System, Case Western Reserve University, Cleveland, OH; i Corewell Health West/Michigan State University; j Westchester Medical Centre, Valhalla, NY.

**Keywords:** CDC WONDER, Disparities, Mortality, Obstructive sleep apnea, Respiratory failure, Trends

## Abstract

Obstructive sleep apnea (OSA) and respiratory failure (RF) represent increasing clinical and public health challenges in the United States, yet national trends in mortality remain underexplored. This study evaluates trends in mortality associated with OSA and RF to highlight targeted prevention and management across demographics and regions. Using the CDC WONDER multiple cause of death (MCD) database, we retrieved death certificates from 1999 to 2023 to analyze mortality related to OSA and RF among adults aged ≥ 25 years. Age-adjusted mortality rates (AAMRs) per 100,000 were calculated and categorized by demographics and region. Joinpoint regression was used to estimate annual percent change (APC) and average APC. A total of 71,202 deaths occurred due to OSA and RF. The AAMR observed a significant and steep incline from 0.25 in 1999 to 2.26 in 2023 (AAPC: 9.3; 95% CI: 8.04 to 10.65, *P* < .01). Men had higher AAMRs than women (men: 0.32; women: 0.21) in 1999 to (men: 2.79; women: 1.85) in 2023. Racially, NH Blacks demonstrated the highest increase in AAMR from 0.53 in 1999 to 2.16 in 2023.The overall AAMR remained higher in nonmetropolitan areas compared to metropolitan areas (1.23 vs 1.01). Regionally, the Midwest recorded the greatest burden with AAMR increasing from 0.21 in 1999 to 2.55 in 2023. State-level AAMRs ranged from 0.58 (New York) to 1.92 (Nebraska) in 2020. Using nationwide retrospective data from 1999 to 2023, our study revealed that adults with concomitant OSA and RF face steadily rising mortality rates, with marked differences observed across demographic groups and geographic regions in the U.S., underscoring the importance of tailored strategies such as expanding access to early detection, and ensuring equitable healthcare delivery to reduce mortality and improve outcomes among such patients, particularly in disproportionately affected populations and regions.

## 1. Introduction:

Obstructive sleep apnea (OSA) is one of the most underdiagnosed diseases worldwide and is strongly associated with adverse health outcomes and increased morbidity.^[1,2]^ Recent estimates suggest that approximately 936 million adults aged 30–69 years are living with OSA worldwide, with the highest prevalence observed in North and South America.^[2]^ A large-scale United States (U.S.) study reported that 26% of individuals aged 30–70 had at least mild OSA, while up to 90% of men and 78% of women over the age of 70 exhibited mild forms of the condition.^[2]^ In the U.S. alone, 26 million adults have been diagnosed with OSA, and this number is projected to rise to 76 million by 2050, although a significant proportion still remains undiagnosed.^[3]^ Moreover, as a multifactorial disorder with substantial social consequences, OSA has placed a considerable economic burden, estimated at US$12.4 billion in 2015.^[4]^ The most important risk factor and primary determinant of severity in the development and progression of OSA is obesity. Additional factors associated with increased risk include male sex, advanced age, craniofacial structural abnormalities, positive family history, cigarette smoking, and the use of alcohol or sedative agents.

Respiratory failure is a life-threatening condition that occurs when the lungs fail to adequately exchange gases, ultimately leading to high mortality rates.^[5]^ Between 2002 and 2017, the national adult annual incidence of respiratory failure in the U.S. increased by 197%, rising from 429 to 1275 cases per 100,000 adults.^[6]^ Respiratory failure may present in both acute and chronic forms. A systematic analysis of the Global Burden of Disease reported that in 2017, 544.9 million individuals worldwide were living with chronic respiratory diseases; a substantial increase compared to 1990 estimates with a global prevalence of 7.1%.^[5]^ Each year, approximately 4 million people die prematurely due to chronic respiratory failure, while acute respiratory failure accounts for an additional 2.6 million deaths approximately. Furthermore, a U.S. based study identified 1,434,349 acute respiratory failure related deaths, with mortality rates rising from 74.9 per 100,000 in 2014 to 85.6 per 100.000 in 2018.^[7]^

OSA is characterized by reduced tonic activity of the pharyngeal muscles during sleep, causing recurrent upper airway collapse and increased airflow resistance.^[8–10]^ These repetitive obstructions lead to intermittent hypoxemia and hypercapnia, which stimulate arousals to restore airway patency.^[11]^ Over time, the recurrent cycles of hypoventilation and arousal impair ventilatory control, blunt chemoreceptor sensitivity to carbon dioxide, and promote chronic retention of CO_2_. Consequently, patients with OSA may progress from episodic nocturnal hypoventilation to chronic hypercapnic respiratory failure, now recognized as a significant cause of morbidity and mortality.^[12–14]^ Obesity, sedative use, and coexisting pulmonary diseases such as COPD further worsen this trajectory by amplifying hypoxemia, hypercapnia, and pulmonary hypertension. Thus, OSA serves not only as a sleep-related breathing disorder but also as an important precursor to respiratory failure, contributing substantially to cardiopulmonary complications and mortality. Although the presence of OSA in patients with diagnosed respiratory failure has been increasingly recognized as a significant, yet frequently underappreciated, contributor to mortality.

More than two-thirds of patients presenting with hypercapnia in acute care settings have underlying OSA when tested, yet only 10–25% are diagnosed in routine clinical practice. The increasing coexistence of OSA and respiratory failure underscores the urgent need to better understand their complex relationship in order to optimize patient care and treatment strategies. This study aims to address that gap by providing a comprehensive analysis of OSA-related mortality among patients with respiratory failure in the U.S. between 1999 and 2023. Closing this knowledge gap is essential, as sleep apnea is a treatable condition, and hypercapnic respiratory failure remains a common disorder with high morbidity and mortality. Furthermore, this review explores racial disparities in comorbidity and mortality, along with temporal trends and differences by sex, geographic region, and urban–rural settings, using data from the CDC WONDER database.

## 2. Methods

### 2.1. Study design

We conducted a retrospective analysis using death certificate data retrieved from the Centers for Disease Control and Prevention Wide-Ranging Online Data for Epidemiologic Research (CDC WONDER) Multiple cause of Death database and analyzed data for adults aged 25 and older between 1999 and 2023 to assess long term temporal trends in mortality related to OSA and respiratory failure, using the International Statistical Classification of Diseases and Related Health Problems-10^th^ Revision (ICD-10) as follows: G47.3 for OSA and J96 for respiratory failure. These ICD codes have been previously used to identify OSA and respiratory failure individually.^[15–18]^ For the purpose of analysis, deaths were included only when both OSA and RF were mentioned on the death certificate, either as the underlying or multiple cause of death. Death certificates where only OSA or only RF was listed were excluded from analysis. This study was exempt from Institutional review board (IRB) approval as it used publicly available, deidentified data and adheres to the STROBE (Strengthening the Reporting of Observational Studies in Epidemiology) guidelines for reporting.^[19]^

### 2.2. Data abstraction

Mortality counts and population estimates for OSA and RF-related deaths were stratified year and demographics such as sex, race, region and states was extracted. Racial and ethnic categories were recorded on death certificates and classified as Hispanic or Latino, non-Hispanic (NH) white, NH Black or African American, NH American Indian or Alaskan Native, and NH Asian or Pacific Islander. The National Center for Health Statistics Urban-Rural Classification Scheme was used to assess the population by urban (metropolitan area had [population ≥ 1 million], and rural (population < 50,000) counties per the 2013 U.S. census classification which is available only for the period 1999 to 2020, because AAMRs provided after 2020 in the CDC WONDER database were deemed unreliable, hence data beyond that year were omitted.^[20]^ Intermediate county classifications were not included in the primary analytic cohort. Regions were stratified into Northeast, Midwest, South, and West according to the U.S. Census Bureau definitions.^[21]^ Place of death was categorized into medical facilities, hospice, home and nursing Home/Long-Term care facilities. Similar epidemiologic investigations examining national mortality patterns have employed this same approach using CDC WONDER data, demonstrating its reliability for capturing temporal trends and demographic disparities in large populations.^[22–24]^

### 2.3. Statistical analysis

Age adjusted mortality rates (AAMRs) per 100,000 population were computed from 1999 to 2023 by year, sex, race/ethnicity, state, and from 1999 to 2020 for urban-rural status with 95% CIs were calculated, standardized to the 2000 U.S. population, using the direct method.^[25]^ Joinpoint Regression Program (Joinpoint V 5.4.0.0, National Cancer Institute) was used to determine the annual percent change (APC) with 95% CI in AAMR to quantify national annual trends and outcomes in OSA and respiratory failure-related mortality.^[26]^ This method allows identification of significant changes in AAMR over time by fitting log-linear regression models where temporal variation occurred. Variation in APCs were considered if the slope describing the change in mortality was significantly different from zero using two tailed t testing. Average annual percentage changes (AAPC) with 95% CIs were computed to summarize overall mortality trends over the course of the study. A *P*-value of <.05 indicated statistical significance.

## 3. Results

Between 1999 and 2023, a total of 71,202 deaths occurred due to concomitant OSA and RF among adults aged > 25 years in the U.S. Information regarding location of death were available for 71,200 cases. Of these, 65.13% occurred in medical facility, 18.65% in decedent’s home, 10.49% in nursing homes/long term care, and 3.82% in hospice setting (Tables S1 and S2, Supplemental Digital Content 1**).**

### 3.1. Annual trends

**Figure 1.**
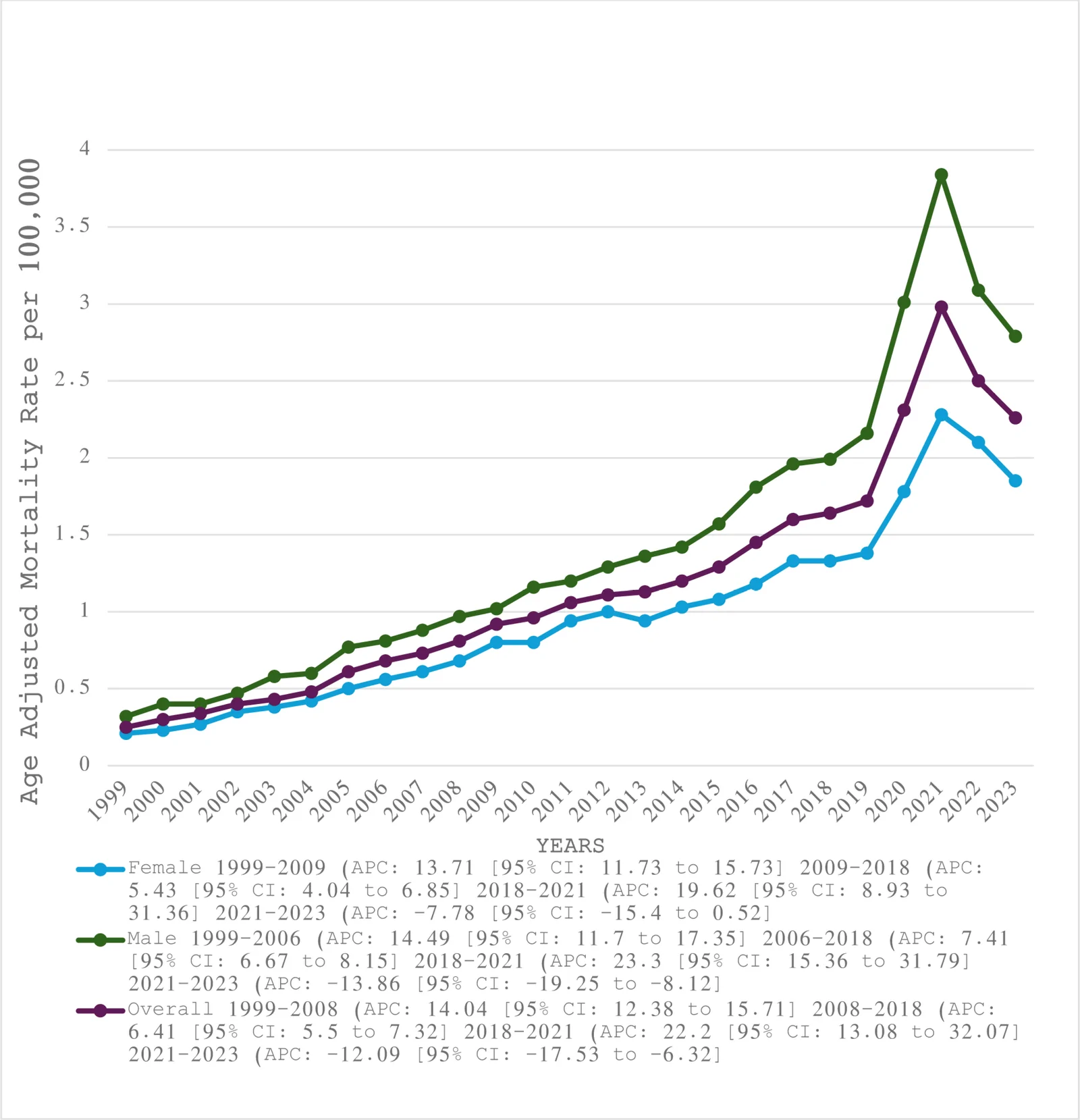
Age-adjusted mortality rates (AAMR) from OSA and respiratory failure in the US by gender and overall (1999–2023).

The AAMR observed an overall marked rise during the study timeframe, from 0.25 (95% CI: 0.23–0.28) in 1999 to 2.26 (95% CI: 2.21–2.32) in 2023, corresponding to an AAPC of 9.3 (95% CI: 8.04–10.65; *P* < .01). From 1999 to 2008, AAMR exhibited a significant escalation (APC: 14.04; 95% CI: 12.38–15.71; *P* < .01), followed by a steeper rise from 2008 to 2018 (APC: 6.41; 95% CI: 5.5–7.32; *P* < .01). A pronounced surge occurred through 2021 (APC: 22.2; 95% CI: 13.08–32.07; *P* < .01). However, a significant and sharp decline followed from 2021 to 2023 (APC: −12.09; 95% CI: −17.53 to −6.3; *P* < .01) (Fig. 1, Tables S3 and S4, Supplemental Digital Content 2**).**

### 3.2. Sex

Throughout the study period, AAMR was consistently higher among men compared to women increasing from 0.32 in 1999 to 2.79 in 2023, indicating a pronounced rise from 1999 to 2006 (APC: 14.49; 95% CI: 11.7–23.41; *P* < .01), a steep increase from 2006 to 2018 (APC: 7.41; 95% CI: 6.67–8.15; *P* < .01), drastic increase from 2018 to 2021 (APC: 23.3; 95% CI: 15.36–31.79; *P* < .01), and a considerable significant decline until 2023 (APC: −13.86; 95% CI: −19.25 to −8.12; *P* < .01). Among women, AAMR rose from 0.21 in 1999 to 1.85 in 2023, with an initial surge from 1999 to 2009 (APC: 13.71; 95% CI: 11.73–15.73; *P* < .01), followed by a moderate rise through 2018 (APC: 5.43; 95% CI: 4.04–6.85; *P* < .01). An enormous incline occurred through 2021 (APC: 19.62; 95% CI: 8.93–31.36; *P* < .01).This was followed by an insignificant decline until 2023 (APC: −7.7; 95% CI: −15.4 to 0.52; *P* = .063) (Fig. 1, Tables S3 and S4, Supplemental Digital Content 2)

### 3.3. Race/ethnicity

**Figure 2.**
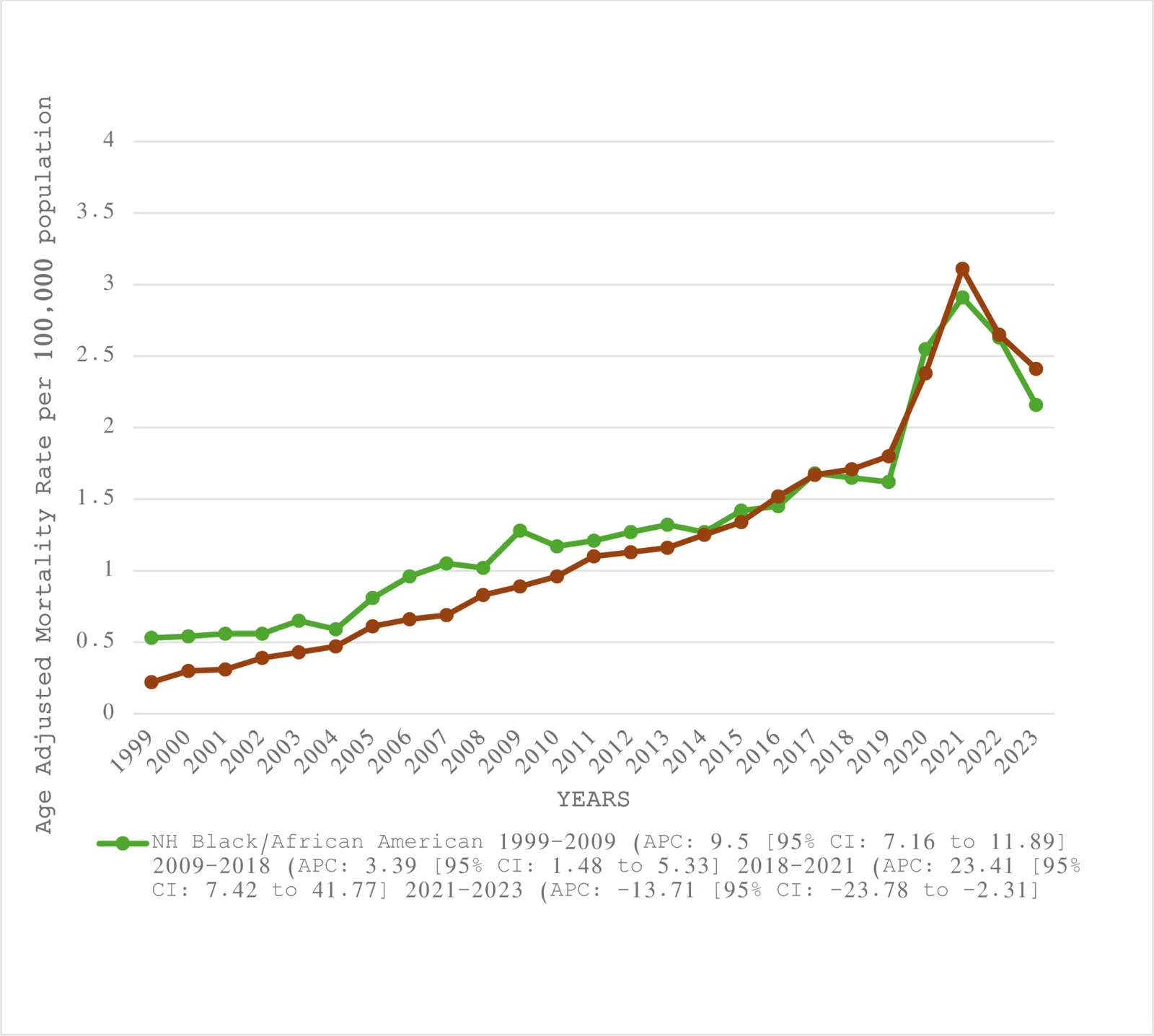
Age-adjusted mortality rates (AAMR) from OSA and respiratory failure in the US by race (1999–2023).

When stratified by race, Non-Hispanic (NH) Black/African American individuals demonstrated the greatest overall AAMR of increasing from 0.53 in 1999 to 2.16 in 2023 relative to NH Whites where AAMR rose from 0.22 in 1999 to 2.41 in 2023. Data for the NH American Indian or Alaska Native population, NH Asian or Pacific Islander population and the Hispanic or Latino population was unreliable, hence excluded (Fig. 2, Tables S3 and S5, Supplemental Digital Content 2**).**

NH Black/African American adults experienced a consistent rise in AAMR from 1999 to 2009 (APC: 9.78; 95% CI: 7.52 to 12.09; *P* < .01). This was followed by a moderate incline from 2009 to 2018 (APC: 3.5; 95% CI: 1.59–5.44; *P* < .01). AAMR reported a sharp escalation from 2018 to 2021 (APC: 23.38; 95% CI: 8.35 to 40.51; *P* < .01), and terminated in 2023 with a decline (APC: −14.3; 95% CI: −23.75 to −3.68; *P* < .01). NH White individuals showed an initial rapid increase in AAMR from 1999 to 2008 (APC: 14.28; 95% CI: 12.83–15.74; *P* < .01), followed by a steep rise from 2008 to 2018 (APC: 7.19; 95% CI: 6.38–8; *P* < .01). A significant and marked incline persisted through 2021 (APC: 21.44; 95% CI: 13.58–29.84; *P* < .01), which was succeeded by a decline from 2021 to 2023 (APC: −9.71; 95% CI: −15.14 to −3.94; *P* < .01).

### 3.4. Geographic regions

#### 3.4.1. Census region

**Figure 3.**
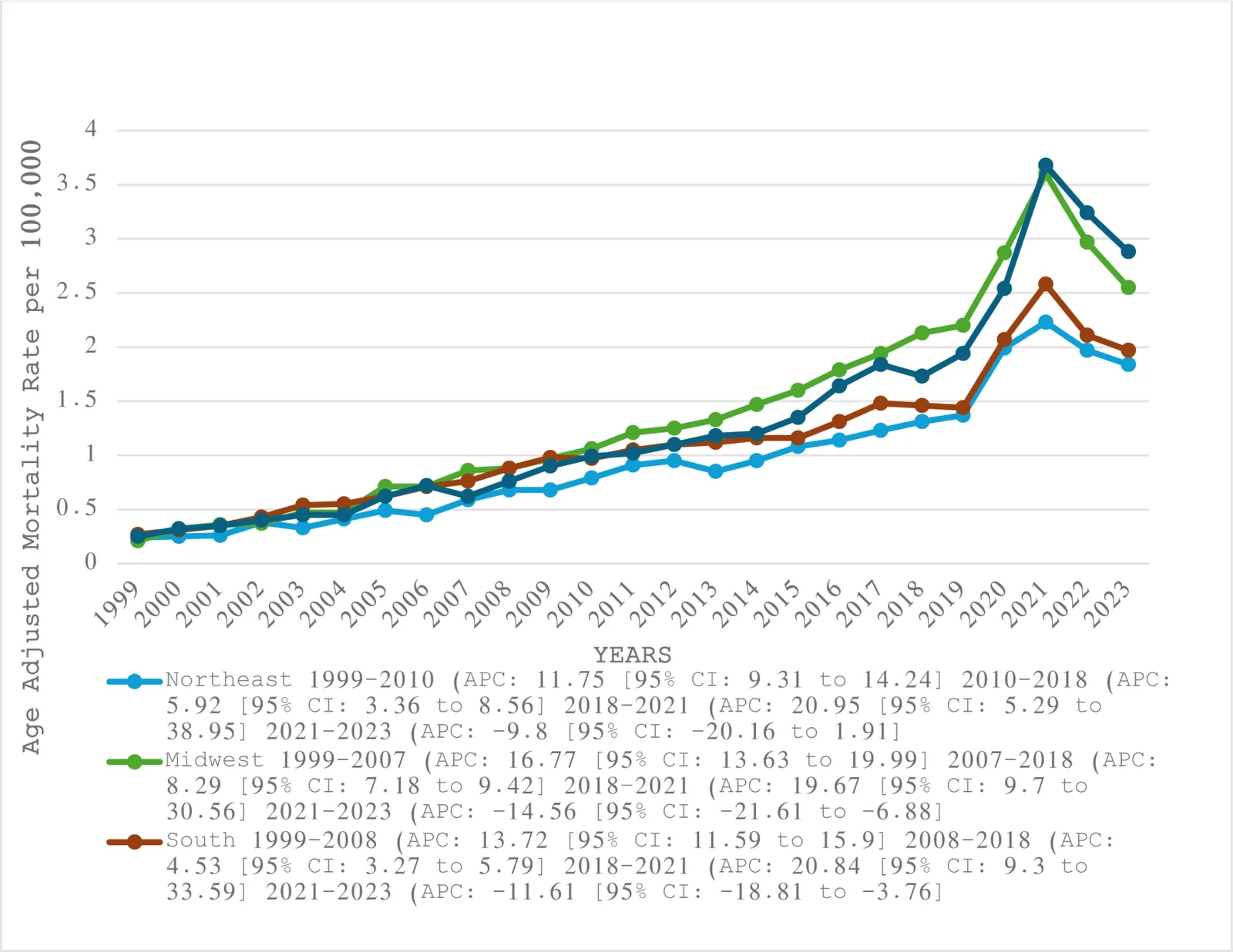
Age-adjusted mortality rates (AAMR) from OSA and respiratory failure in the US by census region (1999–2023).

Throughout the study period, adults living in different U.S. census regions exhibited persistent regional disparities in AAMRs for OSA RF-related mortality. The Midwest reported the highest mortality burden, with AAMR increasing from 0.21 in 1999 to 2.55 in 2023, corresponding to an AAPC of 10.24% (95% CI: 8.59–11.93; *P* < .01). Similarly, the Northeast exhibited an incline from 0.24 in 1999 to 1.84 in 2023, reflecting an AAPC of 8.9% (95% CI: 6.55–11.31; *P* < .01). The AAMR in the South rose from 0.27 in 1999 to 1.97 in 2023, with an AAPC of 8.33% (95% CI: 6.68–10.01; *P* < .01). The Western region demonstrated a comparable upward trend, with AAMR rising from 0.25 in 1999 to 2.88 in 2023, exhibiting an AAPC of 9.46% (95% CI: 7.41–11.54; *P* < .01) (Fig. 3, Table S6, Supplemental Digital Content 3**).**

#### 3.4.2. States

**Figure 4.**
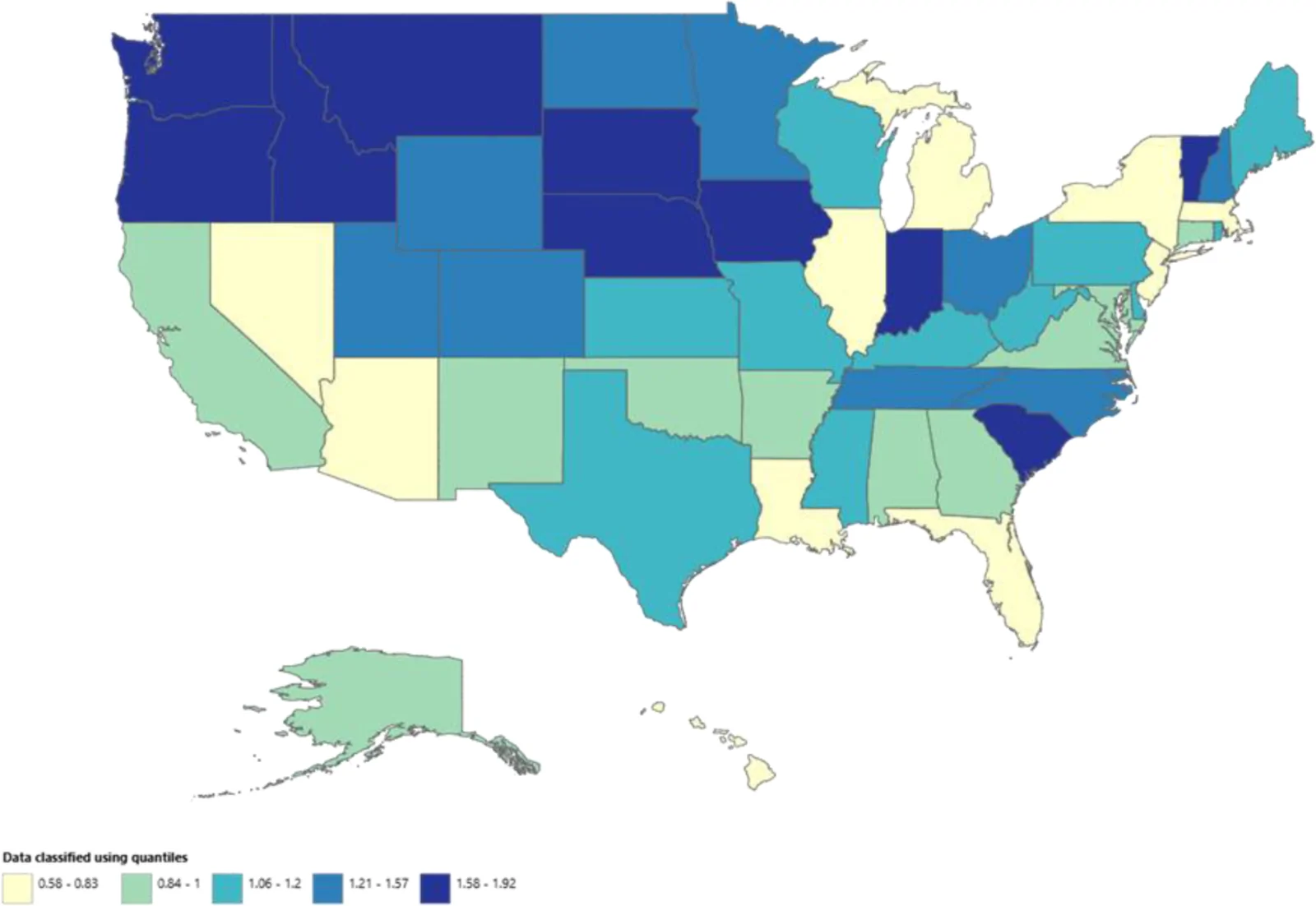
Age-adjusted mortality rates (AAMR) from OSA and respiratory failure in the US by States (1999–2020).

Notable disparities in AAMRs were evident across U.S. states, with AAMRs ranging from 0.58 in New York to 1.92 in Nebraska. States in the top 90th percentile included Nebraska, Montana, South Dakota, Indiana, and Oregon, which had almost triple the AAMR of the states that fell in the lower 10th percentile, such as New York, Louisiana, Massachusetts, Florida, and Nevada (Fig. 4, Table S7, Supplemental Digital Content 4)

#### 3.4.3. Urbanization

**Figure 5.**
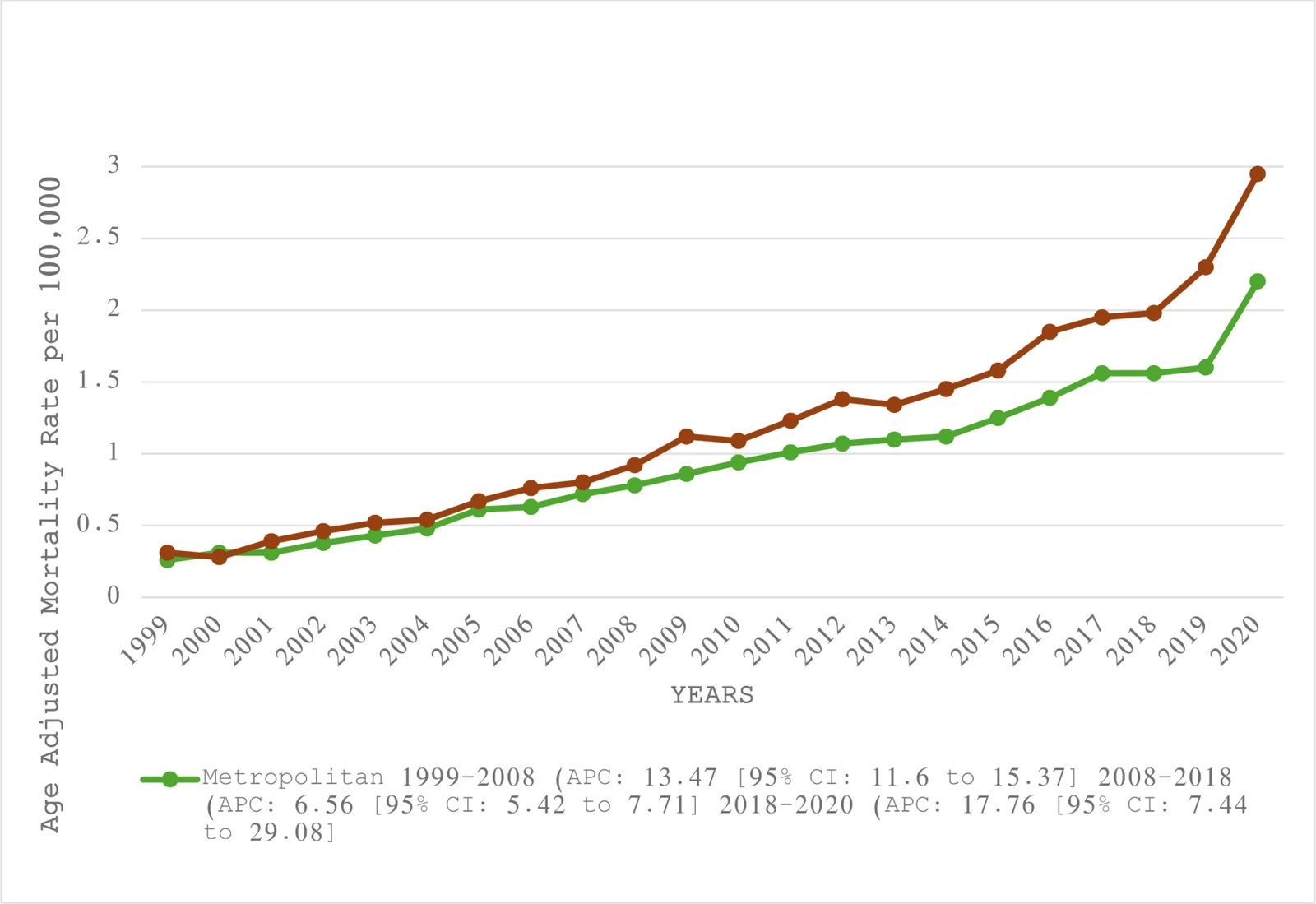
Age-adjusted mortality rates (AAMR) from OSA and respiratory failure in the U.S. by Urbanization (1999–2020).

The overall AAMR remained higher in nonmetropolitan areas 1.23 (95% CI: 1.21–1.26) compared to metropolitan areas 1.01 (95% CI: 1–1.02) throughout the study period (Fig. 5, Tables S3 and S8, Supplemental Digital Content 2**).**

nonmetropolitan areas experienced a significant increase in AAMR from 1999 to 2009 (APC: 13.52; 95% CI: 11.65–15.43; *P* < .01), followed by continued growth from 2009 to 2018 (APC: 7.32; 95% CI: 5.94–8.72; *P* < .01), and an accelerating rise until 2020 (APC: 20.05; 95% CI: 9.36–31.79; *P* < .01).In Metropolitan areas, AAMR rose significantly from 1999 to 2008 (APC: 13.47; 95% CI: 11.6–15.37; *P* < .01), followed by a steep increase from 2008 to 2018 (APC: 6.56; 95% CI: 5.42–7.71; *P* < .01), and a pronounced surge until 2020 (APC: 17.76; 95% CI: 7.44–29.08; *P* < .01).

## 4. Discussion

**Figure 6.**
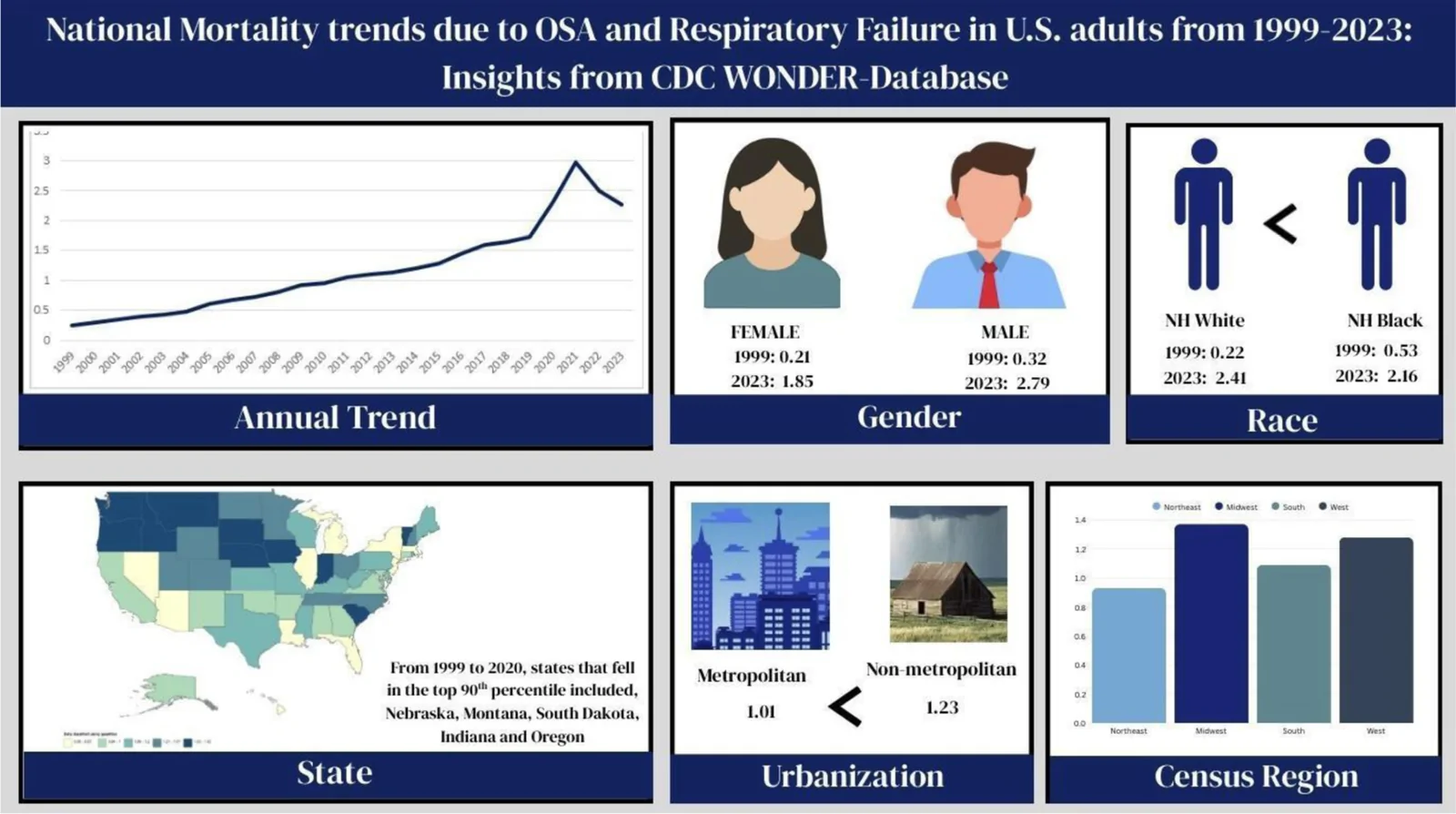
Key findings of our study are demonstrated in the central illustration.

Our study observed an overall increase in AAMR between the years 1999 and 2023, with the majority deaths occurring in medical facilities, followed by decedents’ homes, nursing homes or long-term care facilities, and hospice settings. Mortality trends showed an initial rise from 1999 to 2008, followed by moderate increases to 2018, a pronounced peak during the COVID-19 pandemic in 2021, and a subsequent decline by 2023. Men consistently experienced higher mortality than women, though both sexes demonstrated a simultaneous upward trend over time. Racial disparities were notable, with NH Black adults exhibiting a higher overall AAMR compared with NH White adults, despite a greater absolute number of deaths among Whites. Geographically, the Midwest reported the highest regional AAMR, followed by the West, South, and Northeast. Finally, urbanization patterns indicated that nonmetropolitan areas had persistently higher AAMRs than metropolitan areas, although both settings experienced significant increases over time. (Fig. 6)

The observed upward trend in mortality associated with OSA and respiratory failure may reflect the increasing burden of population-level risk factors such as obesity and aging, as well as the cumulative impact of comorbidities that exacerbate respiratory compromise.^[27,28]^ With the steep rise in obesity rates, the prevalence of Americans suffering from sleep-disordered breathing and chronic hypoventilation has increased significantly.^[29]^ In addition, improvements in diagnostic practices, particularly the expanded use of polysomnography, may have facilitated more frequent identification and reporting of OSA, possibly contributing to the higher recorded incidence of cases.^[22,26,30]^ Concurrently, an aging population and a growing burden of comorbidities, most notably the COPD–OSA overlap syndrome, have been directly associated with greater vulnerability to hypercapnic decompensation.^[31]^ Although positive airway pressure (PAP) therapy remains a highly effective treatment modality for OSA, adherence rates continue to be suboptimal, limiting its overall protective benefits.^[32]^ The sharp increase in mortality observed during 2020–2021 coincided with the COVID-19 pandemic and may reflect, at least in part, the increased vulnerability of individuals with preexisting respiratory disorders to acute infectious illness.^[33]^ Notably, a study involving 44,275 patients with COVID-19 suggested that OSA was associated with a 1.6-fold increased risk of severe disease compared to mild cases. Although mortality was not significantly elevated in this cohort, the increased disease severity may suggest that OSA amplifies respiratory compromise during these acute episodes, consistent with the population-level mortality trends observed.^[34]^ Following 2021, a marked decline in mortality was observed through 2023. National surveillance data reported a 1.2% reduction in deaths from chronic lower respiratory diseases between 2021 and 2022, continuing a downward trend possibly initiated during the pandemic period.^[35]^ An alternative explanation is that the 2020–2021 increase may partly reflect COVID-19-related deaths in which respiratory failure (J96) was documented and OSA was recorded as a comorbidity. Thus, the observed increase should not be interpreted as a causal rise in OSA-related respiratory failure mortality.

This study demonstrates a consistent increase in mortality due to OSA and respiratory failure, particularly in men. Available data report a male-to-female OSA prevalence ratio of 3:1 to 5:1 in the general population.^[36]^ Studies suggest that men typically present with classic symptoms such as loud snoring and witnessed apneas, facilitating earlier recognition, whereas women more often report nonspecific complaints like fatigue, insomnia, and daytime sleepiness and are diagnosed later in life, often at a higher BMI, potentially leading to under- or delayed recognition.^[37]^ Additionally, physiological determinants, including a stronger hypoxic ventilatory response and greater upper airway collapsibility in men, likely predispose them to unstable breathing and more severe disease.^[38]^ Similar trends are observed for respiratory failure, where men show a higher mortality burden. Nationwide mortality data from 2014 to 2018 demonstrated greater ARF- and ARDS-related deaths in men, corroborating our results.^[7]^ Similarly, a multicenter cohort study of ICU patients with laboratory-confirmed COVID-19 revealed that despite being younger and having fewer comorbidities, men were at significantly higher risk of respiratory failure and death compared with women.^[39]^ Occupational exposures, reported nearly twice as often by men in the COPDGene study (47.9% vs 20.1%), were similarly associated with airflow obstruction, respiratory symptoms, emphysema, and gas trapping in both sexes, yet the greater exposure among men likely amplifies their overall disease burden.^[40]^ Alongside the physiological and epidemiological factors described, both OSA and respiratory failure emerge as key contributors to an increased pattern of mortality in men.

NH Black adults in our analysis experienced a disproportionately high AAMR burden compared with NH White adults, consistent with broader evidence of racial inequities in sleep health and respiratory outcomes. Several factors have been associated with racial disparities in OSA and respiratory outcomes. These factors include differences in the prevalence of risk factors, diagnosis and treatment patterns, healthcare access, and socioeconomic conditions, although they were not directly assessed in the present study.^[41]^ U.S.-based studies report persistent barriers to OSA diagnosis and treatment, including lower rates of prior diagnosis, reduced access to specialty care, and lower adherence to positive airway pressure (PAP) therapy.^[42]^ For instance, the Jackson Heart Sleep Study found only 4–5% of participants with clinically significant OSA had ever been diagnosed, while the HomePAP trial observed that Black patients used CPAP nearly 92 minutes less nightly than White patients, a gap further exacerbated by extensive CMS compliance requirements.^[43,44]^ According to a review by Fontanez et al., at the time of diagnosis, Black patients, particularly men, often present with more severe disease, highlighted by a higher Apnea–Hypopnea Index (AHI), symptom, and comorbidity burden.^[45]^ Beyond OSA, disparities extend into acute respiratory failure, where Black patients experience greater illness severity at presentation, higher comorbidity burden, and a more advanced disease stage.^[46]^ These outcomes are further compounded by genetic susceptibility, healthcare biases, and socioeconomic barriers, including limited insurance coverage and language access, as proposed by previous studies.^[46]^ During the COVID-19 pandemic, such inequities were magnified, with Black adults experiencing disproportionately larger losses in life expectancy and excess respiratory-related deaths compared with White adults.^[47]^ Similar trends are evident for ARDS, highlighting how systemic, structural, and social determinants of health increase vulnerability across both acute and chronic respiratory conditions, reinforcing the combined burden on Black populations.^[48]^

Our analysis revealed the highest burden of mortality in the Midwest, followed by the West, South, and then Northeast, suggesting a complex interplay of healthcare access, socio-climatic factors, and public health infrastructure. Notably, a national analysis of ARF/ARDS mortality for 2014–2018 reported disproportionately higher AAMRs in Southern states compared with the Midwest.^[7]^ In contrast, mortality data from the National Center for Health Statistics (1999–2019) showed OSA-related deaths to be more frequent in the Midwest, indicating that regional differences may be disease-specific and influenced by comorbidity profiles, lifestyle risk factors, or healthcare delivery patterns.^[49]^ The latest CDC adult obesity maps further identify the Midwest as one of the regions with the highest prevalence (36%), a factor strongly associated with sleep-disordered breathing and hypoventilation that can progress to respiratory failure.^[50]^ Tobacco exposure also varies by state and region, with CDC data showing the Midwest had the highest prevalence of current use in 2021 (22.1%), while continuing to serve as a major determinant of chronic respiratory injury that elevates the risk of acute failure.^[51,52]^ At the state level, higher AAMRs were observed in Montana, South Dakota, and Wyoming compared with lower rates in New York, Hawaii, and Massachusetts. These geographic differences may reflect variation in population characteristics, healthcare resources, environmental exposures, and other unmeasured factors.

Our findings of persistently higher AAMRs in nonmetropolitan areas align with broader evidence demonstrating a widening rural–urban mortality gap over the past two decades, particularly among working-age adults.^[52]^ Similar trends have been reported for ARF and ARDS, where limited availability of critical care resources, such as mechanical ventilation and trained personnel, has been suggested as a factor in the increased mortality rate in nonmetropolitan areas^[7]^ Additionally, lifestyle and behavioral risk factors also contribute to this disparity.^[53]^ Rural populations have consistently higher smoking prevalence, lower quit ratios, and greater nicotine dependence compared to urban populations, compounded by structural barriers such as fewer cessation programs and lower insurance coverage.^[54]^ Obesity, a well-established risk factor for the dual burden, likewise demonstrates a disproportionate impact in rural communities, where prevalence exceeds that of urban counties and is influenced by variations in diet, income, and access to healthcare services.^[55]^ Occupational exposure adds to the vulnerability, with rural workers reporting markedly higher levels of vapor, gas, dust, and fume exposure (VGDF), which are strongly linked to chronic bronchitis and airflow limitation that can precipitate respiratory failure.^[56]^ Moreover, disparities in access to diagnostic and treatment services for sleep disorders remain evident. In the U.S., rural residents with OSA are more likely to remain undiagnosed or present with greater disease severity due to limited access to sleep specialists and diagnostic testing, underscoring a significant care gap.^[57]^ Initiatives such as the VA TeleSleep program demonstrate that expanding telehealth and home testing can substantially increase access to OSA diagnosis and management in rural populations, highlighting both the existing gap and possible solutions.^[58]^ Together, these factors represent potential contributors to the higher AAMRs observed in nonmetropolitan settings, although their individual contributions could not be determined from the present dataset.

Our findings regarding the place of death provide insight into end-of-life care patterns in the US for these disorders. The predominance of hospital-based deaths (over 65%) underscores the acute nature of respiratory decompensation, where patients frequently require advanced ventilatory support.^[59]^ However, the notable proportion of deaths occurring at home or in nursing facilities underscores the chronic as well as the progressive course of these disorders. In OSA specifically, underdiagnosis and suboptimal treatment may cause individuals to be vulnerable to cardiometabolic complications, some of which may manifest outside specialized care settings.^[59]^ Prior evidence has also demonstrated that OSA increases the risk of sudden cardiac death (SCD), nocturnal arrhythmias, and unrecognized mortality during sleep, which may account for a proportion of deaths occurring in other settings.^[60]^ Similarly, respiratory failure often follows exacerbations of chronic lung disease, but the transition to hospice in a minority of cases suggests a recognition of the irreversible decline associated with advanced disease. Taken together, these patterns highlight the dual impact of OSA and respiratory failure, contributing to deaths occurring in both healthcare facilities and community settings.

Integrating artificial intelligence, machine learning, and deep learning techniques to create more precise prediction models for OSA-related negative outcomes and mortality risk would also help future population-based studies. Such techniques could enable better characterization of the complex interplay among demographic, clinical, and healthcare-related variables that may lead to negative results and help to identify high-risk groups.^[61]^

### 4.1. Limitations

Several limitations should be considered when interpreting our findings. First, the reliance on CDC WONDER mortality data means data were derived from death certificates, which are subject to misclassification and underreporting. OSA, in particular, is frequently undiagnosed during life and therefore may be omitted as a contributing cause of death, leading to an underestimation of its original impact. Our analysis could not differentiate between acute vs chronic forms of respiratory failure or interpret disease severity (e.g., hypoxemia indices, polysomnography findings), which limited the ability to assess clinical phenotypes or interactions between OSA and respiratory failure. Additionally, while stratification by sex, race/ethnicity, geography, and urbanization highlights disparities, the database does not provide tailored information on individual-level risk factors such as obesity, smoking status, occupational exposures, or adherence to therapies (e.g., PAP use), all of which may confound mortality patterns. Moreover, the observed peak during the COVID-19 period cannot be distinguished from pandemic-related diagnostic and reporting practices, potentially increasing mortality attributed to respiratory compromise. Our methodology, which implies the use of death certificates, does not establish the accurate cause of death because OSA and RF were both mentioned as contributing causes of mortality on death certificates. Moreover, the COVID-19 pandemic may have contributed to the observed 2020–2021 mortality increase, as respiratory failure (J96) may have been documented in COVID-19 deaths with OSA listed as a comorbidity. Thus, the increase may not represent a direct rise in OSA-related respiratory failure mortality.

### 4.2. Strengths and clinical implications

A major strength of our study lies in its utilization of two decades of nationally representative mortality data from the CDC WONDER database, which provides comprehensive coverage of the U.S. population and ensures robust population-level inferences. By examining over 71,000 deaths where OSA and RF were listed as contributing causes, our analysis encompasses both temporal patterns and sociodemographic disparities. Stratification by sex, race/ethnicity, geography, and urbanization allowed us to highlight vulnerable subgroups, while state-level and place-of-death analyses added specificity. The extended timeframe of 1999–2023 warranted an assessment of evolving trends, including the pandemic-related increase in mortality. Together, these elements strengthen the generalizability and relevance of our findings across diverse U.S. populations.

The observed disparities in OSA and respiratory failure-related mortality carry direct clinical and public health implications. Persistently higher mortality in men, NH Black adults, and residents of rural or Midwestern states underscores the need for targeted strategies to improve screening, diagnosis, and management in these groups. The high prevalence of obesity and tobacco use in regions with elevated mortality highlights the importance of integrating risk factor modification with respiratory care. Moreover, the predominance of hospital-based deaths illustrates the critical deterioration that characterizes these conditions, while the substantial proportion of deaths occurring at home or in nursing facilities reflects missed opportunities for earlier diagnosis and intervention, particularly for OSA. Future population-based studies may also benefit from integrating artificial intelligence, machine learning, and deep learning approaches to develop more refined prediction models for OSA-related adverse outcomes and mortality risk.

### 4.3. Conclusion

Our study highlights that OSA and respiratory failure together constitute a significant and underrecognized contributor to mortality in the United States. Using over two decades of nationally representative CDC data, we observed a steady rise in mortality burden, peaking in a pandemic-related surge, followed by a partial decline in more recent years. These findings emphasize the dynamic nature of respiratory-related deaths and their vulnerability to shifts in disease patterns and external stressors. The analysis also revealed striking demographic and geographic disparities, including consistently higher mortality in men, disproportionate burdens among NH Black adults, and persistently elevated rates in rural and Midwestern populations. Such variation underscores the interplay of biological vulnerability, socioeconomic and systemic inequities in healthcare access. Notably, the predominance of hospital-based deaths reflects the rapid deterioration that characterizes these conditions, while the subset of deaths outside the medical facility illustrates the ongoing gaps in early recognition and long-term management of OSA and respiratory compromise.

## Author contributions

**Conceptualization:** Maryam Saghir.

**Formal analysis:** Maryam Saghir.

**Writing – original draft:** Maryam Saghir, Eshal Saghir, Nida Shoaib, Barka Sajid.

**Data curation:** Nida Shoaib.

**Writing – review & editing:** Muhammad Affan, Hasibullah Aminpoor, Hafsa Shahid, Javeria Shamim Hayat.

**Supervision:** Hasibullah Aminpoor, Syed Saddam Hussain.

**Validation:** Hafsa Shahid, Javeria Shamim Hayat.

















## References

[1] Benjafield AV Ayas NT Eastwood PR. Estimation of the global prevalence and burden of obstructive sleep apnoea: a literature-based analysis. Lancet Respir Med. 2019;7:687–98. 10.1016/S2213-2600(19)30198-5 PMC7007763 31300334

[2] Iannella G Pace A Bellizzi MG. The global burden of obstructive sleep apnea. Diagnostics (Basel). 2025;15:1088. 10.3390/diagnostics15091088 PMC12071658 40361906

[3] Ali D Qureshi S Siddiqui H. Rising cardiovascular mortality among obstructive sleep apnea patients: United States epidemiological trends (1999–2019). Heart Lung. 2025;70:271–7. 10.1016/j.hrtlng.2025.01.005 39798186

[4] Watson NF. Health care savings: the economic value of diagnostic and therapeutic care for obstructive sleep apnea. J Clin Sleep Med. 2016;12:1075–7. 10.5664/jcsm.6034 PMC4957182 27448424

[5] Soriano JB Kendrick PJ Paulson KR. Prevalence and attributable health burden of chronic respiratory diseases, 1990–2017: a systematic analysis for the Global Burden of Disease Study 2017. Lancet Respir Med. 2020;8:585–96. 10.1016/S2213-2600(20)30105-3 PMC7284317 32526187

[6] Kempker JA Abril MK Chen Y Kramer MR Waller LA Martin GS. The Epidemiology of respiratory failure in the United States 2002-2017: a serial cross-sectional study. Crit Care Explorations. 2020;2:e0128. 10.1097/CCE.0000000000000128 PMC7314331 32695994

[7] Parcha V Kalra R Bhatt SP Berra L Arora G Arora P. Trends and geographic variation in acute respiratory failure and ARDS mortality in the United States. Chest. 2021;159:1460–72. 10.1016/j.chest.2020.10.042 PMC7581392 33393472

[8] Cumpston E Chen P. Sleep apnea syndrome. [Updated 2023 Sep 4]. In: StatPearls. StatPearls Publishing; 2026. Available at: https://www.ncbi.nlm.nih.gov/books/NBK564431/33232089

[9] Tangel DJ Mezzanotte WS White DP. Influence of sleep on tensor palatini EMG and upper airway resistance in normal men. J Appl Physiol (1985). 1991;70:2574–81. 10.1152/jappl.1991.70.6.2574 1885452

[10] Tangel DJ Mezzanotte WS Sandberg EJ White DP. Influences of NREM sleep on the activity of tonic vs. inspiratory phasic muscles in normal men. J Appl Physiol (1985). 1992;73:1058–66. 10.1152/jappl.1992.73.3.1058 1400018

[11] Jyothi I Renuka Prasad K Rajalakshmi R Satish Kumar R Ramphanindra T Vijayakumar T. Obstructive sleep apnea: a pathophysiology and pharmacotherapy approach. In: Vats M, ed. Noninvasive Ventilation in Medicine - Recent Updates. IntechOpen; 2019. Available at: https://www.intechopen.com/books/noninvasive-ventilation-in-medicine-recent-updates/obstructive-sleep-apnea-a-pathophysiology-and-pharmacotherapy-approach. Accessed August 8, 2026.

[12] Locke BW Brown JP Sundar KM. The role of obstructive sleep apnea in hypercapnic respiratory failure identified in critical care, inpatient, and outpatient settings. Sleep Med Clin. 2024;19:339–56. 10.1016/j.jsmc.2024.02.012 PMC11068091 38692757

[13] Adler D Janssens JP. The pathophysiology of respiratory failure: control of breathing. Respiration. 2019;97:93–104. 10.1159/000494063 30423557

[14] McNicholas WT Hansson D Schiza S Grote L. Sleep in chronic respiratory disease: COPD and hypoventilation disorders. Eur Respir Rev. 2019;28:190064. 10.1183/16000617.0064-2019 PMC9488904 31554703

[15] Javeid A Kumar D Nawaz J. Rising U.S. mortality associated with coexisting obstructive sleep apnea and obesity, 1999-2019. Sleep Med. 2026;140:108783. 10.1016/j.sleep.2026.108783 41548264

[16] Søgaard M Madsen M Løkke A Hilberg O Sørensen HT Thomsen RW. Incidence and outcomes of patients hospitalized with COPD exacerbation with and without pneumonia. Int J Chron Obstruct Pulmon Dis. 2016;11:455–65. 10.2147/COPD.S96179 PMC4780743 27042038

[17] Sultana R Lam E Hsu E Gurski E Sharma G. 408 Validation of claim based algorithms for sleep apnea using ICD codes. Sleep. 2021;44:A162–A162.

[18] Centers for Disease Control and Prevention. CDC WONDER. CDC; 2026.

[19] Von Elm E Altman DG Egger M Pocock SJ Gøtzsche PC Vandenbroucke JP; STROBE Initiative. The strengthening the reporting of observational studies in epidemiology (STROBE) statement: guidelines for reporting observational studies. J Clin Epidemiol. 2008;61:344–9. 10.1016/j.jclinepi.2007.11.008 18313558

[20] Aggarwal R Chiu N Loccoh EC Kazi DS Yeh RW Wadhera RK. Rural-urban disparities: diabetes, hypertension, heart disease, and stroke mortality among black and white adults, 1999-2018. J Am Coll Cardiol. 2021;77:1480–1. 10.1016/j.jacc.2021.01.032 PMC8210746 33736831

[21] Ingram DD Franco SJ. 2013 NCHS urban-rural classification scheme for counties. Vital Health Stat 2. 2014:1–73. 24776070

[22] Saghir M Affan M Saghir E. Rising dementia mortality among tobacco users in the US, 2005-2020: insights from CDC-WONDER database. Neurodegenerative Dis Manage. 2026;16:1–12. 10.1080/17582024.2026.2647102 41978491

[23] Saghir M Affan M Ahmed AM. Rising mortality and post-COVID shifts due to stroke in adults with dyslipidemia in the U.S. from 1999 to 2023: analysis of the CDC WONDER database. Int J Neurosci. 2026;136:808–22. 10.1080/00207454.2026.2639360 41773527

[24] Saghir M Kritika FNU Ali Baig MM. The silent epidemic: cardiovascular mortality among patients with thyroid disorders-insights from CDC-WONDER database (1999-2023). J Investig Med. 2026:10815589261448842. 10.1177/10815589261448842 42186295

[25] Anderson RN Rosenberg HM. Age standardization of death rates: implementation of the year 2000 standard. Natl Vital Stat Rep. 1998;47:1–20. 9796247

[26] National Cancer Institute. Joinpoint Regression Program, version 2016. Surveillance Research Program. 2016. Bethesda (MD): National Cancer Institute. Available at: https://surveillance.cancer.gov/joinpoint/. Accessed April 16, 2025.

[27] Locke BW Lee JJ Sundar KM. OSA and chronic respiratory disease: mechanisms and epidemiology. Int J Environ Res Public Health. 2022;19:5473. 10.3390/ijerph19095473 PMC9105014 35564882

[28] Rottoli M Bernante P Belvedere A. How important is obesity as a risk factor for respiratory failure, intensive care admission and death in hospitalised COVID-19 patients? Results from a single Italian centre. Eur J Endocrinol. 2020;183:389–97. 10.1530/EJE-20-0541 PMC9494325 32674071

[29] Venkat DG Badr MS. Respiratory disorders and their association with clinical outcomes in COVID-19: a narrative review of current literature. Ann Palliat Med. 2023;12:1232–43. 10.21037/apm-22-1427 37164968

[30] Centers for Disease Control and Prevention. Adult Obesity Facts. CDC; 2024.

[31] Kapur VK Auckley DH Chowdhuri S. Clinical practice guideline for diagnostic testing for adult obstructive sleep apnea: an American academy of sleep medicine clinical practice guideline. J Clin Sleep Med. 2017;13:479–504. 10.5664/jcsm.6506 PMC5337595 28162150

[32] Czerwaty K Dżaman K Sobczyk KM Sikorska KI. The overlap syndrome of obstructive sleep apnea and chronic obstructive pulmonary disease: a systematic review. Biomedicines. 2022;11:16. 10.3390/biomedicines11010016 PMC9856172 36672523

[33] Rotenberg BW Murariu D Pang KP. Trends in CPAP adherence over twenty years of data collection: a flattened curve. J Otolaryngol Head Neck Surg. 2016;45:43. 10.1186/s40463-016-0156-0 PMC4992257 27542595

[34] Nassi-Liberman O Oberman B Strahl T Yosef N Shlomi D. Association between obstructive sleep apnea (OSA) and COVID-19 severity. J Sleep Res. 2025;34:e14260. 10.1111/jsr.14260 PMC11911043 38867140

[35] Kochanek KD Murphy SL Xu J Arias E. Deaths: final data for 2021. Natl Vital Stat Rep. 2024;73:1–139. Available at: https://www.cdc.gov/nchs/products/databriefs/db492.htm. Accessed September 3, 2025. 10.15620/cdc/158787 PMC12051083 40305404

[36] Bonsignore MR Saaresranta T Riha RL. Sex differences in obstructive sleep apnoea. Eur Respir Rev. 2019;28:190030. 10.1183/16000617.0030-2019 PMC9488655 31694839

[37] Lin CM Davidson TM Ancoli-Israel S. Gender differences in obstructive sleep apnea and treatment implications. Sleep Med Rev. 2008;12:481–96. 10.1016/j.smrv.2007.11.003 PMC2642982 18951050

[38] Schwarz EI Schiza S. Sex differences in sleep and sleep-disordered breathing. Curr Opin Pulm Med. 2024;30:593–9. 10.1097/MCP.0000000000001116 PMC11451933 39189037

[39] Toth-Manikowski SM Caldwell J Joo M.; STOP-COVID Investigators. Sex-related differences in mortality, acute kidney injury, and respiratory failure among critically ill patients with COVID-19. Medicine (Baltim). 2021;100:e28302. 10.1097/MD.0000000000028302 PMC8677989 34918709

[40] Marchetti N Garshick E Kinney GL.; COPDGene Investigators. Association between occupational exposure and lung function, respiratory symptoms, and high-resolution computed tomography imaging in COPDGene. Am J Respir Crit Care Med. 2014;190:756–62. 10.1164/rccm.201403-0493OC PMC4299608 25133327

[41] Jackson CL Powell-Wiley TM Gaston SA Andrews MR Tamura K Ramos A. Racial/ethnic disparities in sleep health and potential interventions among women in the United States. J Womens Health (Larchmt). 2020;29:435–42. 10.1089/jwh.2020.8329 PMC7097680 32096683

[42] Agarwal S Monsod P Cho YS. Racial disparity in obstructive sleep apnea care and its impact on cardiovascular health. Curr Sleep Med Rep. 2024;10:414–8. 10.1007/s40675-024-00308-6 PMC11500798 39463890

[43] Johnson DA Guo N Rueschman M Wang R Wilson JG Redline S. Prevalence and correlates of obstructive sleep apnea among African Americans: the Jackson Heart Sleep Study. Sleep. 2018;41:zsy154. 10.1093/sleep/zsy154 PMC6187109 30192958

[44] Wallace DM Shafazand S Aloia MS Wohlgemuth WK. The association of age, insomnia, and self-efficacy with continuous positive airway pressure adherence in black, white, and Hispanic U.S. Veterans. J Clin Sleep Med. 2013;9:885–95. 10.5664/jcsm.2988 PMC3746716 23997701

[45] Acevedo-Fontanez AI Patel SR. Racial disparities in obstructive sleep apnea care in the United States. Sleep. 2025;48:zsaf078. 10.1093/sleep/zsaf078 PMC12163130 40130545

[46] Bime C Poongkunran C Borgstrom M. Racial differences in mortality from severe acute respiratory failure in the United States, 2008-2012. Ann Am Thorac Soc. 2016;13:2184–9. 10.1513/AnnalsATS.201605-359OC PMC7531026 27668888

[47] Aburto JM Tilstra AM Floridi G Dowd JB. Significant impacts of the COVID-19 pandemic on race/ethnic differences in US mortality. Proc Natl Acad Sci U S A. 2022;119:e2205813119. 10.1073/pnas.2205813119 PMC9436308 35998219

[48] Blank JA Armstrong-Hough M Valley TS. Disparities among patients with respiratory failure. Curr Opin Crit Care. 2023;29:493–504. 10.1097/MCC.0000000000001079 PMC10599128 37641499

[49] Lee YC Chang KY Mador MJ. Racial disparity in sleep apnea-related mortality in the United States. Sleep Med. 2022;90:204–13. 10.1016/j.sleep.2021.11.014 35202926

[50] Centers for Disease Control and Prevention. Adult Obesity Prevalence Maps. CDC; 2025.

[51] Holford TR McKay L Jeon J. Smoking histories by state in the U.S. Am J Prev Med. 2023;64:S42–52. 10.1016/j.amepre.2022.08.018 PMC11186469 36653233

[52] Cornelius ME Loretan CG Jamal A. Tobacco product use among adults - United States, 2021. MMWR Morb Mortal Wkly Rep. 2023;72:475–83. 10.15585/mmwr.mm7218a1 PMC10168602 37141154

[53] Cross SH Califf RM Warraich HJ. Rural-urban disparity in mortality in the US from 1999 to 2019. JAMA. 2021;325:2312–4. 10.1001/jama.2021.5334 PMC8188271 34100876

[54] Parker MA Weinberger AH Eggers EM Parker ES Villanti AC. Trends in rural and urban cigarette smoking quit ratios in the US From 2010 to 2020. JAMA Netw Open. 2022;5:e2225326. 10.1001/jamanetworkopen.2022.25326 PMC9350718 35921112

[55] Okobi OE Ajayi OO Okobi TJ. The burden of obesity in the rural adult population of America. Cureus. 2021;13:e15770. 10.7759/cureus.15770 PMC8290986 34295580

[56] Doney BC Henneberger PK Humann MJ Liang X Kelly KM Cox-Ganser JM. Occupational exposure to vapor-gas, dust, and fumes in a cohort of rural adults in iowa compared with a cohort of urban adults. MMWR Surveill Summ. 2017;66:1–5. 10.15585/mmwr.ss6621a1 PMC5829718 29095802

[57] Leonhard AG Donovan LM. First steps toward implementing primary care obstructive sleep apnea management in rural West Virginia. Ann Am Thorac Soc. 2025;22:982–3. 10.1513/AnnalsATS.202505-496ED PMC12254148 40590657

[58] Chun VS Whooley MA Williams K. Veterans Health Administration TeleSleep enterprise-wide initiative 2017–2020: bringing sleep care to our nation’s veterans. J Clin Sleep Med. 2023;19:913–23. 10.5664/jcsm.10488 PMC10152352 36708262

[59] Wunsch H Linde-Zwirble WT Angus DC Hartman ME Milbrandt EB Kahn JM. The epidemiology of mechanical ventilation use in the United States. Crit Care Med. 2010;38:1947–53. 10.1097/CCM.0b013e3181ef4460 20639743

[60] Blackwell JN Walker M Stafford P Estrada S Adabag S Kwon Y. Sleep apnea and sudden cardiac death. Circ Rep. 2019;1:568–74. 10.1253/circrep.CR-19-0085 PMC7083593 32201748

[61] Aliyeva A Hashimli R Yilmaz B. Hematological biomarkers of the obstructive sleep apnea syndrome: a machine learning-based diagnostic and prognostic model. J Clin Med. 2025;14:8437. 10.3390/jcm14238437 PMC12693066 41375740

