# Supplementary material for: National mortality trends due to OSA and respiratory failure in U.S adults from 1999–2023: Insights from the CDC-WONDER database
Source: Medicine (Baltimore). 2026 Sep 18;105(38):e50662. doi: 10.1097/MD.0000000000050662 (PMC13593082; doi:10.1097/MD.0000000000050662)
Supplement: Supplementary file 1 [file medi-105-e50662-s001.docx]

**Supplemental Table 1** OSA and Respiratory Failure–related Deaths, Stratified by Sex and Race, in Adults in the United States, 1999 to 2023.

| Year | Overall | Women | Men | NH White | NH Black or African American |
| --- | --- | --- | --- | --- | --- |
| 1999 | 458 | 195 | 263 | 361 | 90 |
| 2000 | 549 | 237 | 312 | 450 | 93 |
| 2001 | 620 | 281 | 339 | 519 | 99 |
| 2002 | 744 | 355 | 389 | 633 | 105 |
| 2003 | 866 | 403 | 463 | 721 | 129 |
| 2004 | 935 | 443 | 492 | 803 | 120 |
| 2005 | 1,204 | 530 | 674 | 1,024 | 168 |
| 2006 | 1,327 | 605 | 722 | 1,116 | 195 |
| 2007 | 1,478 | 681 | 797 | 1,242 | 217 |
| 2008 | 1,698 | 791 | 907 | 1,456 | 218 |
| 2009 | 1,904 | 918 | 986 | 1,590 | 276 |
| 2010 | 2,089 | 968 | 1,121 | 1,779 | 266 |
| 2011 | 2,314 | 1,131 | 1,183 | 1,996 | 283 |
| 2012 | 2,509 | 1,229 | 1,280 | 2,170 | 301 |
| 2013 | 2,620 | 1,206 | 1,414 | 2,262 | 323 |
| 2014 | 2,834 | 1,335 | 1,499 | 2,461 | 319 |
| 2015 | 3,117 | 1,426 | 1,691 | 2,702 | 365 |
| 2016 | 3,592 | 1,589 | 2,003 | 3,153 | 380 |
| 2017 | 4,083 | 1,854 | 2,229 | 3,541 | 459 |
| 2018 | 4,232 | 1,895 | 2,337 | 3,672 | 464 |
| 2019 | 4,518 | 1,976 | 2,542 | 3,962 | 466 |
| 2020 | 6,242 | 2,632 | 3,610 | 5,347 | 758 |
| 2021 | 7,962 | 3,308 | 4,654 | 6,847 | 875 |
| 2022 | 6,928 | 3,125 | 3,803 | 5,995 | 759 |
| 2023 | 6,379 | 2,843 | 3,536 | 5,544 | 665 |
| Total | 71,202 | 31,956 | 39,246 | 61,346 | 8,393 |

OSA, Obstructive sleep apnea; NH, non-Hispanic
