# Supplementary material for: National mortality trends due to OSA and respiratory failure in U.S adults from 1999–2023: Insights from the CDC-WONDER database
Source: Medicine (Baltimore). 2026 Sep 18;105(38):e50662. doi: 10.1097/MD.0000000000050662 (PMC13593082; doi:10.1097/MD.0000000000050662)
Supplement: Supplementary file 2 [file medi-105-e50662-s002.docx]

**Supplemental Table 3** Annual percent change (APC) of OSA and Respiratory Failure–related Age-Adjusted Mortality Rates per 100,000 in Adults in the United States, 1999 to 2023.

| Year Interval | APC (95% CI) |
| --- | --- |
| Overall |  |
| 1999-2008 | 14.04 (12.38 to 15.71) |
| 2008-2018 | 6.41 (5.5 to 7.32) |
| 2018-2021 | 22.2 (13.08 to 32.07) |
| 2021-2023 | −12.09 (−17.53 to −6.3) |
| Women |  |
| 1999-2009 | 13.71 (11.73 to 15.73) |
| 2009-2018 | 5.43 (4.04 to 6.85) |
| 2018-2021 | 19.62 (8.93 to 31.36) |
| 2021-2023 | −7.78 (−15.4 to 0.52) |
| Men |  |
| 1999-2006 | 14.49 (11.7 to 17.35) |
| 2006-2018 | 7.41 (6.67 to 8.15) |
| 2018-2021 | 23.3 (15.36 to 31.79) |
| 2021-2023 | −13.86 (−19.25 to −8.12) |
| NH Black or African American | |
| 1999-2009 | 9.78 (7.52 to 12.09) |
| 2009-2018 | 3.5 (1.59 to 5.44) |
| 2018-2021 | 23.38 (8.35 to 40.51) |
| 2021-2023 | −14.3 (−23.75 to −3.68) |
| NH White |  |
| 1999-2008 | 14.28 (12.83 to 15.74) |
| 2008-2018 | 7.19 (6.38 to 8) |
| 2018-2021 | 21.44 (13.58 to 29.84) |
| 2021-2023 | −9.71 (−15.14 to −3.94) |
| Metropolitan |  |
| 1999-2008 | 13.47 (11.6 to 15.37) |
| 2008-2018 | 6.56 (5.42 to 7.71) |
| 2018-2020 | 17.76 (7.44 to 29.08) |
| Non-metropolitan |  |
| 1999-2009 | 13.52 (11.65 to 15.43) |
| 2009-2018 | 7.32 (5.94 to 8.72) |
| 2018-2020 | 20.05 (9.36 to 31.79) |

APC = annual percent change; NH = non-Hispanic; OSA, Obstructive sleep apnea
