# Supplementary material for: National mortality trends due to OSA and respiratory failure in U.S adults from 1999–2023: Insights from the CDC-WONDER database
Source: Medicine (Baltimore). 2026 Sep 18;105(38):e50662. doi: 10.1097/MD.0000000000050662 (PMC13593082; doi:10.1097/MD.0000000000050662)
Supplement: Supplementary file 3 [file medi-105-e50662-s003.docx]

**Supplemental Table 6** OSA and Respiratory Failure–related Age-Adjusted Mortality Rates per 100,000, Stratified by Census Region in Adults in the United States, 1999 to 2023.

| Census Region | Year | Age Adjusted Rate |
| --- | --- | --- |
| Northeast | 1999 | 0.24 (0.19-0.3) |
| Northeast | 2000 | 0.25 (0.2-0.31) |
| Northeast | 2001 | 0.26 (0.21-0.32) |
| Northeast | 2002 | 0.38 (0.32-0.44) |
| Northeast | 2003 | 0.33 (0.27-0.39) |
| Northeast | 2004 | 0.41 (0.35-0.48) |
| Northeast | 2005 | 0.49 (0.42-0.56) |
| Northeast | 2006 | 0.45 (0.39-0.52) |
| Northeast | 2007 | 0.59 (0.52-0.67) |
| Northeast | 2008 | 0.68 (0.6-0.76) |
| Northeast | 2009 | 0.68 (0.6-0.77) |
| Northeast | 2010 | 0.79 (0.71-0.88) |
| Northeast | 2011 | 0.91 (0.82-1.01) |
| Northeast | 2012 | 0.95 (0.86-1.05) |
| Northeast | 2013 | 0.85 (0.76-0.93) |
| Northeast | 2014 | 0.95 (0.86-1.04) |
| Northeast | 2015 | 1.08 (0.98-1.18) |
| Northeast | 2016 | 1.14 (1.04-1.24) |
| Northeast | 2017 | 1.23 (1.12-1.33) |
| Northeast | 2018 | 1.31 (1.21-1.42) |
| Northeast | 2019 | 1.37 (1.27-1.48) |
| Northeast | 2020 | 1.99 (1.86-2.12) |
| Northeast | 2021 | 2.23 (2.09-2.36) |
| Northeast | 2022 | 1.97 (1.85-2.1) |
| Northeast | 2023 | 1.84 (1.72-1.96) |
| Midwest | 1999 | 0.21 (0.17-0.26) |
| Midwest | 2000 | 0.32 (0.27-0.37) |
| Midwest | 2001 | 0.36 (0.3-0.41) |
| Midwest | 2002 | 0.37 (0.31-0.43) |
| Midwest | 2003 | 0.47 (0.41-0.54) |
| Midwest | 2004 | 0.47 (0.41-0.54) |
| Midwest | 2005 | 0.71 (0.63-0.79) |
| Midwest | 2006 | 0.71 (0.63-0.79) |
| Midwest | 2007 | 0.86 (0.77-0.95) |
| Midwest | 2008 | 0.88 (0.79-0.96) |
| Midwest | 2009 | 0.97 (0.88-1.06) |
| Midwest | 2010 | 1.06 (0.97-1.16) |
| Midwest | 2011 | 1.21 (1.11-1.31) |
| Midwest | 2012 | 1.25 (1.15-1.35) |
| Midwest | 2013 | 1.33 (1.23-1.43) |
| Midwest | 2014 | 1.47 (1.36-1.57) |
| Midwest | 2015 | 1.6 (1.49-1.71) |
| Midwest | 2016 | 1.79 (1.67-1.9) |
| Midwest | 2017 | 1.94 (1.82-2.06) |
| Midwest | 2018 | 2.13 (2.01-2.26) |
| Midwest | 2019 | 2.2 (2.08-2.33) |
| Midwest | 2020 | 2.87 (2.73-3.01) |
| Midwest | 2021 | 3.6 (3.44-3.76) |
| Midwest | 2022 | 2.97 (2.82-3.11) |
| Midwest | 2023 | 2.55 (2.42-2.68) |
| South | 1999 | 0.27 (0.23-0.31) |
| South | 2000 | 0.31 (0.27-0.36) |
| South | 2001 | 0.35 (0.3-0.39) |
| South | 2002 | 0.43 (0.38-0.48) |
| South | 2003 | 0.54 (0.48-0.59) |
| South | 2004 | 0.55 (0.5-0.61) |
| South | 2005 | 0.62 (0.56-0.68) |
| South | 2006 | 0.71 (0.65-0.78) |
| South | 2007 | 0.76 (0.69-0.82) |
| South | 2008 | 0.88 (0.82-0.95) |
| South | 2009 | 0.98 (0.91-1.05) |
| South | 2010 | 0.97 (0.9-1.04) |
| South | 2011 | 1.05 (0.98-1.12) |
| South | 2012 | 1.1 (1.03-1.17) |
| South | 2013 | 1.12 (1.05-1.19) |
| South | 2014 | 1.16 (1.09-1.23) |
| South | 2015 | 1.16 (1.09-1.23) |
| South | 2016 | 1.31 (1.24-1.39) |
| South | 2017 | 1.48 (1.4-1.56) |
| South | 2018 | 1.46 (1.38-1.54) |
| South | 2019 | 1.44 (1.36-1.51) |
| South | 2020 | 2.07 (1.98-2.17) |
| South | 2021 | 2.58 (2.48-2.68) |
| South | 2022 | 2.11 (2.02-2.2) |
| South | 2023 | 1.97 (1.88-2.05) |
| West | 1999 | 0.25 (0.2-0.31) |
| West | 2000 | 0.32 (0.26-0.37) |
| West | 2001 | 0.35 (0.29-0.41) |
| West | 2002 | 0.4 (0.34-0.47) |
| West | 2003 | 0.45 (0.39-0.52) |
| West | 2004 | 0.45 (0.38-0.51) |
| West | 2005 | 0.62 (0.54-0.69) |
| West | 2006 | 0.72 (0.64-0.8) |
| West | 2007 | 0.62 (0.54-0.69) |
| West | 2008 | 0.76 (0.67-0.84) |
| West | 2009 | 0.9 (0.81-0.99) |
| West | 2010 | 0.99 (0.89-1.08) |
| West | 2011 | 1.02 (0.93-1.11) |
| West | 2012 | 1.1 (1.01-1.2) |
| West | 2013 | 1.18 (1.08-1.27) |
| West | 2014 | 1.2 (1.11-1.3) |
| West | 2015 | 1.35 (1.25-1.45) |
| West | 2016 | 1.64 (1.53-1.74) |
| West | 2017 | 1.84 (1.73-1.96) |
| West | 2018 | 1.73 (1.63-1.84) |
| West | 2019 | 1.94 (1.83-2.05) |
| West | 2020 | 2.54 (2.41-2.67) |
| West | 2021 | 3.68 (3.53-3.84) |
| West | 2022 | 3.24 (3.09-3.38) |
| West | 2023 | 2.88 (2.75-3.02) |

OSA, Obstructive sleep apnea
