# Supplementary material for: National mortality trends due to OSA and respiratory failure in U.S adults from 1999–2023: Insights from the CDC-WONDER database
Source: Medicine (Baltimore). 2026 Sep 18;105(38):e50662. doi: 10.1097/MD.0000000000050662 (PMC13593082; doi:10.1097/MD.0000000000050662)
Supplement: Supplementary file 4 [file medi-105-e50662-s004.docx]

**Supplemental Table 7** OSA and Respiratory Failure–related Age-Adjusted Mortality Rates per 100,000, Stratified by States in Adults in the United States, 1999 to 2020.

| State | Age Adjusted Rate |
| --- | --- |
| Alabama | 1.43 (1.27-1.59) |
| Alaska | 1.76 (1.24-2.42) |
| Arizona | 1.46 (1.32-1.59) |
| Arkansas | 1.67 (1.44-1.89) |
| California | 1.62 (1.56-1.69) |
| Colorado | 4 (3.73-4.27) |
| Connecticut | 1.18 (1.02-1.35) |
| Delaware | 1.55 (1.21-1.94) |
| District of Columbia | 1.26 (0.86-1.8) |
| Florida | 1.1 (1.04-1.16) |
| Georgia | 1.17 (1.07-1.28) |
| Hawaii | 1.03 (0.8-1.31) |
| Idaho | 3.19 (2.78-3.6) |
| Illinois | 1.26 (1.17-1.36) |
| Indiana | 2.29 (2.12-2.46) |
| Iowa | 2.96 (2.68-3.25) |
| Kansas | 2.09 (1.83-2.34) |
| Kentucky | 2.15 (1.94-2.36) |
| Louisiana | 1.37 (1.2-1.54) |
| Maine | 1.29 (1.05-1.57) |
| Maryland | 2.18 (2-2.37) |
| Massachusetts | 1.14 (1.02-1.26) |
| Michigan | 1.22 (1.11-1.32) |
| Minnesota | 2.99 (2.77-3.21) |
| Mississippi | 1.95 (1.7-2.2) |
| Missouri | 1.84 (1.68-2) |
| Montana | 5.23 (4.5-5.89) |
| Nebraska | 3.88 (3.45-4.31) |
| Nevada | 1.28 (1.08-1.48) |
| New Hampshire | 1.97 (1.63-2.31) |
| New Jersey | 1.22 (1.11-1.33) |
| New Mexico | 1.45 (1.2-1.69) |
| New York | 1.01 (0.94-1.07) |
| North Carolina | 1.97 (1.84-2.1) |
| North Dakota | 2.33 (1.81-2.94) |
| Ohio | 2.31 (2.18-2.44) |
| Oklahoma | 1.32 (1.14-1.49) |
| Oregon | 3.99 (3.7-4.28) |
| Pennsylvania | 2.23 (2.11-2.35) |
| Rhode Island | 1.52 (1.2-1.89) |
| South Carolina | 2.59 (2.38-2.8) |
| South Dakota | 5.16 (4.43-5.89) |
| Tennessee | 2.41 (2.23-2.59) |
| Texas | 1.71 (1.63-1.8) |
| Utah | 3.08 (2.73-3.43) |
| Vermont | 2.73 (2.19-3.34) |
| Virginia | 1.43 (1.31-1.56) |
| Washington | 3.21 (3.01-3.41) |
| West Virginia | 1.52 (1.27-1.77) |
| Wisconsin | 2.83 (2.62-3.04) |
| Wyoming | 3.93 (3.17-4.81) |

OSA, Obstructive sleep apnea
