# Supplementary material for: National mortality trends due to OSA and respiratory failure in U.S adults from 1999–2023: Insights from the CDC-WONDER database
Source: Medicine (Baltimore). 2026 Sep 18;105(38):e50662. doi: 10.1097/MD.0000000000050662 (PMC13593082; doi:10.1097/MD.0000000000050662)
Supplement: Supplementary file 5 [file medi-105-e50662-s005.docx]

**Supplemental Table 2** OSA and Respiratory Failure –related Mortality, Stratified by Place of Death in Adults in the United States, 1999 to 2023.

| Place of Death | Deaths |
| --- | --- |
| Medical Facility - Inpatient | 43,064 |
| Medical Facility - Outpatient or ER | 3,095 |
| Medical Facility - Dead on Arrival | 206 |
| Medical Facility - Status unknown | 11 |
| Decedent's home | 13,283 |
| Hospice facility | 2,722 |
| Nursing home/long term care | 7,473 |
| Other | 1,239 |
| Place of death unknown | 107 |
| Total | 71,200 |

OSA, Obstructive sleep apnea
