# Supplementary material for: National mortality trends due to OSA and respiratory failure in U.S adults from 1999–2023: Insights from the CDC-WONDER database
Source: Medicine (Baltimore). 2026 Sep 18;105(38):e50662. doi: 10.1097/MD.0000000000050662 (PMC13593082; doi:10.1097/MD.0000000000050662)
Supplement: Supplementary file 6 [file medi-105-e50662-s006.docx]

**Supplemental Table 4** Overall and Sex‐Stratified OSA and Respiratory Failure–related Age-Adjusted Mortality Rates per 100,000 in Adults in the United States, 1999 to 2023.

| Year | Overall | Women | Men |
| --- | --- | --- | --- |
| 1999 | 0.25 (0.23-0.28) | 0.21 (0.18-0.24) | 0.32 (0.28-0.36) |
| 2000 | 0.3 (0.28-0.33) | 0.23 (0.2-0.26) | 0.4 (0.35-0.44) |
| 2001 | 0.34 (0.32-0.37) | 0.27 (0.24-0.3) | 0.4 (0.36-0.45) |
| 2002 | 0.4 (0.37-0.43) | 0.35 (0.31-0.39) | 0.47 (0.42-0.52) |
| 2003 | 0.43 (0.4-0.46) | 0.38 (0.34-0.42) | 0.58 (0.53-0.64) |
| 2004 | 0.48 (0.44-0.51) | 0.42 (0.38-0.46) | 0.6 (0.55-0.66) |
| 2005 | 0.61 (0.57-0.64) | 0.5 (0.45-0.54) | 0.77 (0.71-0.83) |
| 2006 | 0.68 (0.64-0.71) | 0.56 (0.51-0.6) | 0.81 (0.75-0.87) |
| 2007 | 0.73 (0.69-0.77) | 0.61 (0.56-0.66) | 0.88 (0.82-0.94) |
| 2008 | 0.81 (0.77-0.85) | 0.68 (0.63-0.73) | 0.97 (0.91-1.04) |
| 2009 | 0.92 (0.88-0.96) | 0.8 (0.75-0.85) | 1.02 (0.96-1.09) |
| 2010 | 0.96 (0.92-1) | 0.8 (0.75-0.85) | 1.16 (1.09-1.23) |
| 2011 | 1.06 (1.02-1.11) | 0.94 (0.89-1) | 1.2 (1.13-1.27) |
| 2012 | 1.11 (1.06-1.15) | 1 (0.94-1.05) | 1.29 (1.22-1.36) |
| 2013 | 1.13 (1.09-1.17) | 0.94 (0.89-0.99) | 1.36 (1.28-1.43) |
| 2014 | 1.2 (1.15-1.24) | 1.03 (0.98-1.09) | 1.42 (1.35-1.49) |
| 2015 | 1.2 (1.25-1.34) | 1.08 (1.02-1.14) | 1.57 (1.49-1.65) |
| 2016 | 1.45 (1.4-1.5) | 1.18 (1.12-1.24) | 1.81 (1.73-1.9) |
| 2017 | 1.6 (1.55-1.65) | 1.33 (1.27-1.4) | 1.96 (1.88-2.04) |
| 2018 | 1.64 (1.59-1.69) | 1.33 (1.27-1.4) | 1.99 (1.91-2.07) |
| 2019 | 1.72 (1.67-1.77) | 1.38 (1.32-1.44) | 2.16 (2.07-2.24) |
| 2020 | 2.31 (2.26-2.37) | 1.78 (1.71-1.85) | 3.01 (2.91-3.11) |
| 2021 | 2.98 (2.92-3.05) | 2.28 (2.2-2.36) | 3.84 (3.73-3.96) |
| 2022 | 2.5 (2.44-2.56) | 2.1 (2.03-2.18) | 3.09 (2.99-3.19) |
| 2023 | 2.26 (2.21-2.32) | 1.85 (1.79-1.92) | 2.79 (2.7-2.89) |

OSA, Obstructive sleep apnea
