# Supplementary material for: National mortality trends due to OSA and respiratory failure in U.S adults from 1999–2023: Insights from the CDC-WONDER database
Source: Medicine (Baltimore). 2026 Sep 18;105(38):e50662. doi: 10.1097/MD.0000000000050662 (PMC13593082; doi:10.1097/MD.0000000000050662)
Supplement: Supplementary file 7 [file medi-105-e50662-s007.docx]

**Supplemental Table 5** OSA and Respiratory Failure–related Age-Adjusted Mortality Rates per 100,000, Stratified by Race in Adults in the United States, 1999 to 2023.

| **Year** | **NH Black or African American** | **NH White** |
| --- | --- | --- |
| 1999 | 0.55 (0.44-0.68) | 0.25 (0.22-0.28) |
| 2000 | 0.54 (0.43-0.66) | 0.31 (0.28-0.34) |
| 2001 | 0.55 (0.44-0.67) | 0.33 (0.3-0.36) |
| 2002 | 0.56 (0.45-0.67) | 0.41 (0.38-0.45) |
| 2003 | 0.68 (0.56-0.79) | 0.43 (0.4-0.46) |
| 2004 | 0.61 (0.49-0.72) | 0.49 (0.46-0.53) |
| 2005 | 0.85 (0.71-0.98) | 0.62 (0.58-0.66) |
| 2006 | 0.98 (0.84-1.12) | 0.68 (0.64-0.73) |
| 2007 | 1.06 (0.91-1.2) | 0.74 (0.69-0.78) |
| 2008 | 1.03 (0.89-1.17) | 0.85 (0.8-0.89) |
| 2009 | 1.33 (1.16-1.49) | 0.93 (0.88-0.98) |
| 2010 | 1.21 (1.06-1.36) | 0.98 (0.93-1.02) |
| 2011 | 1.27 (1.12-1.42) | 1.12 (1.07-1.17) |
| 2012 | 1.31 (1.16-1.47) | 1.19 (1.14-1.24) |
| 2013 | 1.38 (1.22-1.53) | 1.24 (1.18-1.29) |
| 2014 | 1.34 (1.19-1.49) | 1.29 (1.23-1.34) |
| 2015 | 1.47 (1.31-1.63) | 1.4 (1.35-1.46) |
| 2016 | 1.51 (1.36-1.67) | 1.6 (1.54-1.65) |
| 2017 | 1.73 (1.56-1.89) | 1.75 (1.69-1.81) |
| 2018 | 1.75 (1.59-1.92) | 1.78 (1.71-1.84) |
| 2019 | 1.69 (1.53-1.85) | 1.88 (1.82-1.95) |
| 2020 | 2.64 (2.45-2.84) | 2.45 (2.38-2.52) |
| 2021 | 3.06 (2.85-3.27) | 3.28 (3.19-3.36) |
| 2022 | 2.73 (2.53-2.93) | 2.79 (2.71-2.86) |
| 2023 | 2.23 (2.05-2.41) | 2.57 (2.5-2.64) |

OSA, Obstructive sleep apnea; NH, non-Hispanic
