# Supplementary material for: National mortality trends due to OSA and respiratory failure in U.S adults from 1999–2023: Insights from the CDC-WONDER database
Source: Medicine (Baltimore). 2026 Sep 18;105(38):e50662. doi: 10.1097/MD.0000000000050662 (PMC13593082; doi:10.1097/MD.0000000000050662)
Supplement: Supplementary file 8 [file medi-105-e50662-s008.docx]

**Supplemental Table 8** OSA and Respiratory Failure–related Age-Adjusted Mortality Rates per 100,000, Stratified by Urban-Rural Classification in Adults in the United States, 1999 to 2020.

| Year | Metropolitan | Non-metropolitan |
| --- | --- | --- |
| 1999 | 0.26 (0.23-0.29) | 0.31 (0.24-0.38) |
| 2000 | 0.31 (0.28-0.34) | 0.28 (0.22-0.34) |
| 2001 | 0.31 (0.28-0.34) | 0.39 (0.32-0.45) |
| 2002 | 0.38 (0.35-0.41) | 0.46 (0.39-0.53) |
| 2003 | 0.43 (0.4-0.47) | 0.52 (0.44-0.59) |
| 2004 | 0.48 (0.45-0.51) | 0.54 (0.46-0.62) |
| 2005 | 0.61 (0.57-0.65) | 0.67 (0.58-0.75) |
| 2006 | 0.63 (0.59-0.67) | 0.76 (0.67-0.85) |
| 2007 | 0.72 (0.68-0.76) | 0.8 (0.7-0.89) |
| 2008 | 0.78 (0.73-0.82) | 0.92 (0.82-1.02) |
| 2009 | 0.86 (0.82-0.9) | 1.12 (1-1.23) |
| 2010 | 0.94 (0.89-0.99) | 1.09 (0.98-1.19) |
| 2011 | 1.01 (0.96-1.05) | 1.23 (1.12-1.35) |
| 2012 | 1.07 (1.02-1.12) | 1.38 (1.26-1.5) |
| 2013 | 1.1 (1.05-1.15) | 1.34 (1.22-1.46) |
| 2014 | 1.12 (1.07-1.17) | 1.45 (1.33-1.56) |
| 2015 | 1.25 (1.2-1.3) | 1.58 (1.45-1.71) |
| 2016 | 1.39 (1.34-1.44) | 1.85 (1.71-1.98) |
| 2017 | 1.56 (1.51-1.62) | 1.95 (1.81-2.09) |
| 2018 | 1.56 (1.51-1.61) | 1.98 (1.84-2.12) |
| 2019 | 1.6 (1.54-1.65) | 2.3 (2.15-2.44) |
| 2020 | 2.2 (2.14-2.27) | 2.95 (2.78-3.12) |

OSA, Obstructive sleep apnea
